# Using space lidar to infer bubble cloud depth on a global scale

**DOI:** 10.1038/s41598-024-75551-6

**Published:** 2024-10-18

**Authors:** Damien Josset, Stephanie Cayula, Magdalena Anguelova, W. Erick Rogers, David Wang

**Affiliations:** 1 Ocean Sciences Division, U.S. Naval Research Laboratory, NASA Stennis Space Center, John C. Stennis Space Center, MS 39529 USA; 2 Remote Sensing Division, U.S. Naval Research Laboratory, Washington, DC 20375 USA

**Keywords:** Physical oceanography, Applied optics

## Abstract

Visible and microwave satellite measurements can provide the global whitecap fraction. The bubble clouds are three-dimensional structures, and a space-based lidar can provide complementary observations of the bubble depth. Here, we use lidar measurements of the Cloud-Aerosol Lidar and Infrared Pathfinder Satellite Observation (CALIPSO) satellite to quantify global bubble depth from the depolarization. The relationship between CALIPSO bubble depth and wind speed from the Advanced Microwave Scanning Radiometer for EOS (AMSR-E) and AMSR2 is similar to a recently derived relationship based on buoy measurements. The CALIPSO-based bubble depth data show global distributions and seasonal variations consistent with the high wind speed (> 7 m/s) but with some variance. We also found similarities between the CALIPSO bubble depth and the whitecap fraction from AMSR2 and WindSat. Our findings support the use of spaceborne lidar measurements for advancing the understanding of the 3D bubble properties, and the ocean physics at high wind speeds.

## Introduction

Measurements of the whitecap fraction *W*—defined as the fraction of the ocean surface covered by whitecaps (sea foam)—are usually extracted from photographs and video images collected from ships and aircraft^1–4^. More recently, the measurement of whitecaps fraction using passive remote sensing has been demonstrated^5^.

The whitecap fraction *W* quantifies wave breaking with air entrainment. Therefore, *W* is a suitable forcing parameter for developing parameterizations of surface fluxes in models that represent the enhancement of the air-sea interactions due to breaking waves, including heat transfer^6^, gas exchange^7^, and sea spray production^8^. Large-scale wave breaking affects the wave field evolution; thus, its realistic and accurate representation in wave models is a necessity. In this sense, the capability to measure *W* from space is a breakthrough for global observations of air-sea processes.

While oceanic whitecaps are the most visible and direct surface expression of wave breaking, they also mark areas of bubble plumes formed beneath the breaking waves. The bubble plumes are three-dimensional structures with properties described with a range of variables such as void fraction, size distributions, penetration depth, surface area, and bubble plume volumes, often averaged over many wave periods. This complexity implies that quantifying all the statistics of these bubble plumes is essential to obtain robust parameterizations of the fluxes at the ocean-atmosphere interface. Recent studies attest to this by showing that the statistics of bubble plumes are strongly correlated with total wave-breaking dissipation^9–13^. Even if the whitecap fraction and the bubble depths are related^13^, the considerable variation of sea states and breaking wave scales requires additional research to validate such relationships across the global ocean^11^. For this reason, the capability to determine the bubble depth from space combined with the existing capability to determine the whitecap fraction would be a breakthrough for global physical oceanography. It would allow us to confirm the existence of a general whitecaps-to-bubble depth relationship and, in any case, to measure the energy dissipation rate associated with breaking waves^11^.

Lidar systems have been deployed on ships and aircrafts to characterize ocean optical properties, phytoplankton, zooplankton, and fish stocks^14–18^. As active sensors, lidar measurements have distinct advantages over passive remote sensing for ocean observations in that they can provide information on the vertical structure of ocean properties.

Regarding the spatial structure of bubble properties, lidar measurements cannot match the coverage of the passive systems. However, space lidar can provide the average bubble depth, which, in conjunction with passive measurements, provides independent assessments of the bubble volume and the breaking wave energy dissipation rate.

Currently, there are no ocean space lidar systems. However, the National Aeronautics and Space Administration (NASA) and the Centre National d’Etudes Spatiales launched the Cloud-Aerosol Lidar and Infrared Pathfinder Satellite Observation (CALIPSO) satellite in 2006 as part of the A-train Earth Observing Sensor suite^19,20^. The primary instrument on CALIPSO is the Cloud-Aerosol Lidar with Orthogonal Polarization (CALIOP) sensor, and it collected global lidar measurements from 2006 to 2023. Because of its polarization characterization capabilities, the CALIPSO mission offered a unique opportunity for the first global evaluation of bubble properties from a space lidar. Here, we focus on retrieving the bubble depth using CALIOP’s 532 nm polarization channels.

A lidar with depolarization can provide unique information on two bubble properties: the bubble depth^21^ and the void fraction^21,22^. We focus here on the bubble depth because its link with the integrated depolarization is direct (see addendum, Method M1).

The CALIPSO capability for water column profiling is limited because the lidar sensor is designed for atmospheric research and has a coarse in-water vertical resolution of 22.4 m. Therefore, our analysis focuses on the vertically integrated depolarization from 60 m above to 300 m below the ocean surface. As an initial validation of our approach, we compare CALIPSO-based bubble depth data with previously derived wind speed relationships.

## Data and methods

### CALIPSO lidar data analysis

The addendum (Method M1, sections a and b) contains the details of the CALIPSO lidar data analysis and the derivation of the associated uncertainties.

**Fig. 1:**
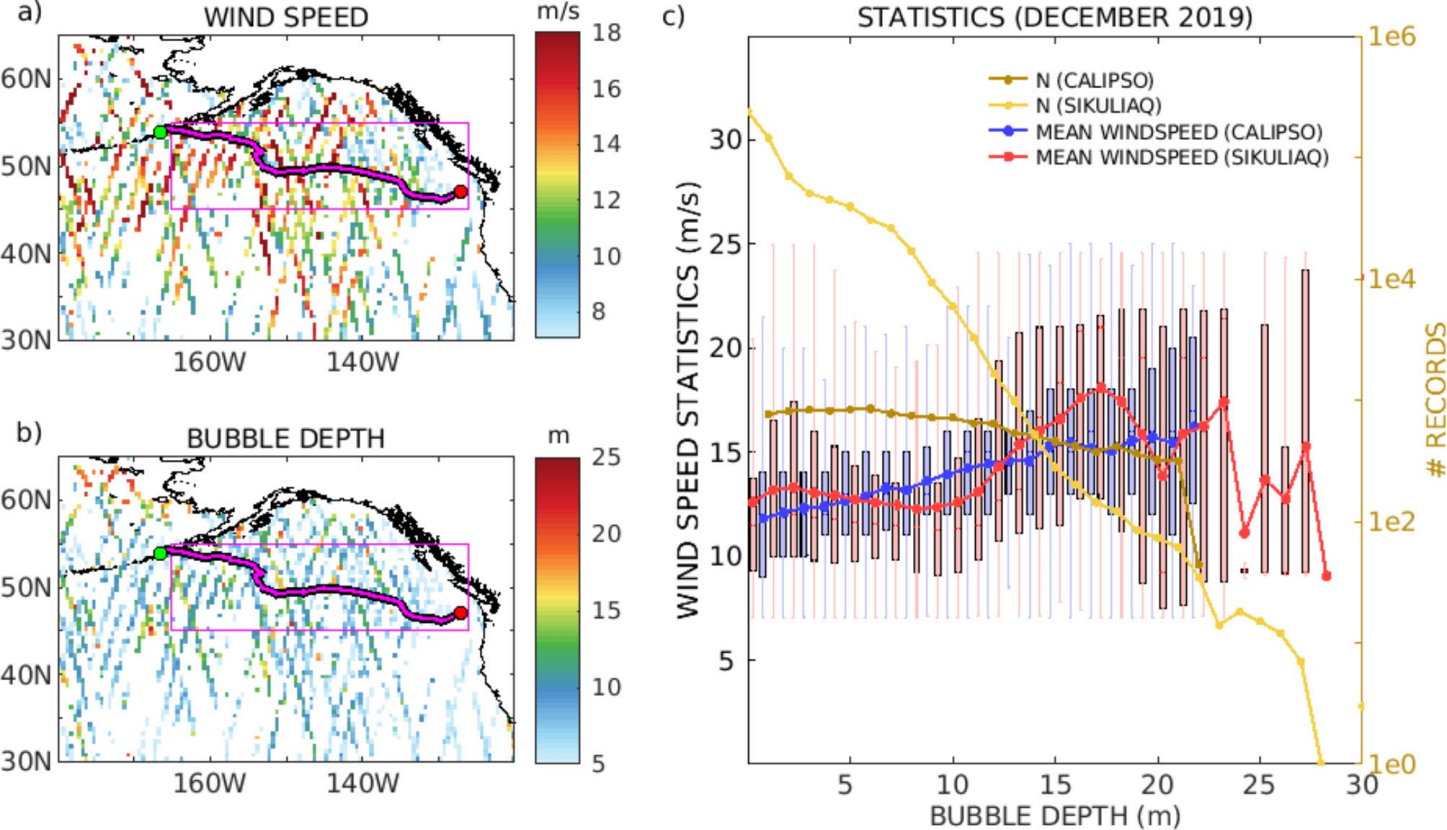
(a) AMSR-E/AMSR2 wind speed collocated with the CALIPSO track in December 2019 during the breaking bubble experiment. (b) Same as a) for the CALIPSO bubble depth. The trajectory of the R/V Sikuliaq is shown in magenta. (c) Boxplot of bubble depth from the NRL shipboard lidar (red) and CALIPSO bubble depth (blue) in December 2019 in the Gulf of Alaska. Only wind speed > 7 m/s are used in the analysis. The vertical box is the interquartile range (IQR), and shows the first (Q1), second (red bar) and third (Q3) quantiles. The whiskers on each boxplot show the limits between (Q1-1.5 X IQR and Q3 + 1.5 X IQR). The number of occurrences is also indicated for the NRL shipboard lidar (yellow) and CALIPSO (dark yellow) with an y-axis on the right.

In support of developing satellite lidar retrievals of bubble depth, Naval Research Laboratory (NRL) deployed a shipboard lidar system^23,24^ during the National Science Foundation (NSF) Breaking Bubbles deployment. For the shipboard lidar, the track is well defined (Fig. 1, left), and the period is limited to the 2019 cruise in the Gulf of Alaska (Breaking Bubbles) between December 4th (Dutch Harbor) and December 23rd (Seattle)^13^). For CALIPSO, to capture sufficient data statistics, we initially limited the coverage to the Gulf of Alaska surrounding the ship track during December 2019 (pink rectangle in Fig. 1, left).

The lidar system acquired 113 h of ocean backscatter profiles during storm conditions, providing high-resolution data to derive a relationship between the bubble depth and lidar depolarization. This dataset can be, in turn, combined with the high-resolution co-polarized observations to determine the bubble void fraction^21,22^. The shipboard lidar high-resolution observations can also be integrated and thus provide the link between the bubble depth and integrated depolarization observations. Any low-resolution lidar system, such as the CALIPSO lidar, can use this relationship.

Assessment of ocean bubble properties from a lidar co-polarization channel is challenging in actual ocean conditions^21^ because of the difficulty in discriminating between the bubble plume and the natural ocean background in this channel. The difficulty increases for a space lidar because the signal-to-noise ratio and the vertical resolution are low.

As wind speed increases, however, the ocean signal measured by CALIPSO’s integrated depolarization is almost entirely due to backscatter from the underwater bubbles. Based on our shipboard lidar measurements taken in the Gulf of Alaska, we found that there is a simple statistical relationship between the shipboard lidar integrated depolarization and the bubble depth derived directly from the lidar high-resolution profiling capabilities^21^.

Therefore, we used the depolarization ratio, defined as the surface integrated cross-polarized backscatter divided by the integrated co-polarized backscatter, to retrieve the bubble depth. This ratio is independent of atmospheric transmittance and is very accurately calibrated. To account for the lack of bubbles at low wind speeds, we included only a subset of the data in the analyses, which was more representative of breaking wave conditions. Specifically, we removed the retrievals under the conditions of very high surface depolarization (> 0.1, to remove sea ice), low wind (< 7 m/s), bubble depth ≤ 0 m (so that the bubbleless ocean does not dominate the statistic), and scenes with liquid water clouds, ice clouds, and stratospheric features (see Method M1).

The CALIPSO data are the Synergized Optical Depth of Aerosols (SODA) product on the ICARE website (https://www.icare.univ-lille.fr/soda/). This product has pre-processed the collocation between CALIPSO and the Advanced Microwave Scanning Radiometer (AMSR) unified data, including AMSR for Earth Observing System (AMSR-E, 2002 to 2011) and AMSR2 (2012 to present). This allowed the pairing of the CALIPSO surface-integrated attenuated backscatter with wind speed retrievals from AMSR-E/AMSR2 measurements^25^. The SODA scientific datasets that we used are the “Perpendicular_Attenuated_Surface_Cross_Section_532”.

and the “Total_Attenuated_Surface_Cross_Section_532”. This data comes directly from the level 1 backscatter signal of the CALIPSO lidar (CAL_LID_L1). The total backscatter and perpendicular backscatter are vertically integrated from 60 m above to 300 m below the ocean surface. A simple combination of these two parameters is used to determine δ_R_ (Eq. 1 in Method M1).

For the current analysis, and as described in Method M1, the relationship between bubble depth and depolarization δ_R_ is:

1 $$\text{Bubble Depth} = \:-20.18{\delta\:}_{R}^{-0.255}+58.47 \text{m}$$

The relationship between depolarization and bubble depth depends on the amount of cross-talk, and multiple scattering. CALIPSO is keeping a higher amount of multiple scattered light than the shipboard lidar, and the distribution of depolarization is shifted towards higher values (Method M1, Fig. M.2). For the data in the Gulf of Alaska, the median depolarization of the shipboard observations is 0.0196 ± 0.0054 and 0.0329 ± 0.0205 for CALIPSO.

The anticipated error budget is described in Method M1. This high depolarization distribution is likely the cause of the high bias that we determined. The use of a rescaling factor for the depolarization to bubble depth relationship could increase the accuracy of the algorithm, by correcting the effects from cross-talk and multiple scattering.

### Whitecap fraction satellite retrievals

Satellite retrievals of whitecap fraction *W* are based on the high emissivity of the sea foam at microwave frequencies measured by radiometers in terms of brightness temperature *T*_*B*_^5^. The whitecap retrieval algorithm utilizes a radiative transfer model to simulate the emissivity of foam-free and foam-covered ocean areas at the top of the atmosphere. Geophysical variables, including wind vector (speed *U*_10_ and direction *φ*), sea surface temperature (*T*), columnar water vapor (*V*), and columnar cloud liquid water (*L*), serve as inputs to the models comprising the whitecap algorithm. The input data (*U*_10_, *φ*, *T*, *V*, *L*) can be taken either from a geophysical model or from satellites. Comparison of different versions of the whitecap retrieval algorithm have shown that the optimal approach is to use *T*_*B*_ and input variables (*U*_10_, *φ*, *T*, *V*, *L*) from the same spaceborne sensor to minimize errors due to time-space collocations.

Physically based whitecap retrievals (as opposed to an initial empirical version) were first developed within the framework of the WindSat mission^26,27^. Recently, the whitecap retrieval algorithm was adapted for use with AMSR2 observations of *T*_*B*_^28^. Modifications were necessary because AMSR2 sensor tracks the WindSat frequency channels (from 6 to 37 GHz) closely but differs in the incidence angles and calibration.

The whitecap algorithm produces *W* retrievals at different microwave frequencies (e.g., 10, 18 and 37 GHz) and two polarizations (horizontal H and vertical V, equivalents of the co-polarization and cross-polarization of lidar observations - Method M1). The *W* values at different frequencies are useful approximations for the thickness of the whitecaps. For example, *W* at 10 GHz represent predominantly thick foam layers formed during the active wave breaking with bubble plumes below featuring large penetration depths and wide range of bubble sizes. In contrast, *W* at 37 GHz includes both thick active whitecaps and thin layers of decaying foam left behind the breaking waves. The polarization differences can be used as a measure for the whitecap sensitivity to wind speed forcing — stronger at H polarization and weaker for V polarization.

In this study, we have used *W* retrievals from AMSR2 and WindSat for 5–22 December 2019 obtained with the respective whitecap algorithms for each sensor. WindSat *T*_*B*_ data are stored at NRL, while AMSR2 *T*_*B*_ data were taken from the Japan Aerospace Exploration Agency (JAXA). We have used AMSR2 Level-1R product, which spatially matches the center positions and footprint sizes for each frequency channel^29^. Before running the whitecap algorithm, the geophysical input data (*U*_10_, *φ*, *T*, *V*, *L*) were retrieved for each sensor. The WindSat *W* retrievals are for a footprint of approximately 25 km × 35 km; these were subsequently grided at 1/4° × 1/4° grid cell. The AMSR2 *W* retrievals are used at their footprint resolution of 14 km × 22 km (no gridding was applied). For both instruments, the *W* values used are averages of the *W* retrievals at 18 GHz, H and V polarizations.

## Results and discussion

### Comparison of the shipboard and space lidar bubble depth data in the Gulf of Alaska

We compared the occurrences of the lidar bubble depth retrievals (NRL shipboard lidar and CALIPSO) as a function of wind speed (Fig. 1, c). Over the 19-day period of breaking bubble field measurements over the whole Gulf of Alaska, the R/V Sikuliaq’s track (Fig. 1, a and b) transected stormy ocean environments. Thus, the shipboard bubble depth values range widely, from 0.14 to 30.93 m, with a median of 1.64 m and a standard deviation of 2.8 m (Fig. 1c). For CALIPSO, the bubble depth range has a minimum of 1.08 m, a maximum of 22.17 m, a median of 9.01 m, and a standard deviation of 5.68 m.

Figure 1 shows that the bubble depth of CALIPSO increases as the wind increases, as expected. However, the horizontal and vertical spatial resolutions of the ship-based measurements are far finer than that achieved with an 80 m diameter CALIPSO footprint. In other words, the NRL shipboard lidar provides several profiles of bubble depth for each bubble cloud. This statistically increases the occurrence of data with a low bubble depth for any wind speed. The maximum or average value of the bubble depth is the quantity increasing as a function of wind speed. CALIPSO does not provide such high-resolution observations and measures one depth for each laser shot for an 80 m diameter cylinder (Method M1, Fig. M.4). This quantity correlates more directly with wind speed than high-resolution observations. Therefore, it is possible to obtain a better correlation with wind speed from the shipboard lidar by averaging the bubble depth values within a given time interval.

Even with the differences in observing geometries and resolution, interesting similarities exist between the bubble depth statistic of the shipboard and spaceborne lidar data. Half of the bubble depth statistic is below 9 m, and the number of bubble observations above 20 m decreases sharply.

There is a positive bias of 4.5 m in the CALIPSO bubble depth. We describe the derivation of this bias in Method M1. A bias correction would bring the CALIPSO data closer to the shipboard lidar data for a bubble depth of around 15 m and above. However, further analysis is required before implementation. Note that the observations of the shipboard lidar do not cover as wide of a meteorological variability as CALIPSO. The shipboard dataset is much more limited in time and space. The swell was extremely steep for some observations of the shipboard lidar. This may change the relationship between wind speed and bubble depth. Therefore, modifying the algorithm based solely on this comparison seems premature.

Interestingly, the linear increase of CALIPSO bubble depth with wind speed is similar to the relationship found by^30^. This is notable given that the algorithm is only based on depolarization and does not use the wind speed dependence explicitly.

### Regional and global comparison of the space lidar bubble depth and wind speed

**Fig. 2:**
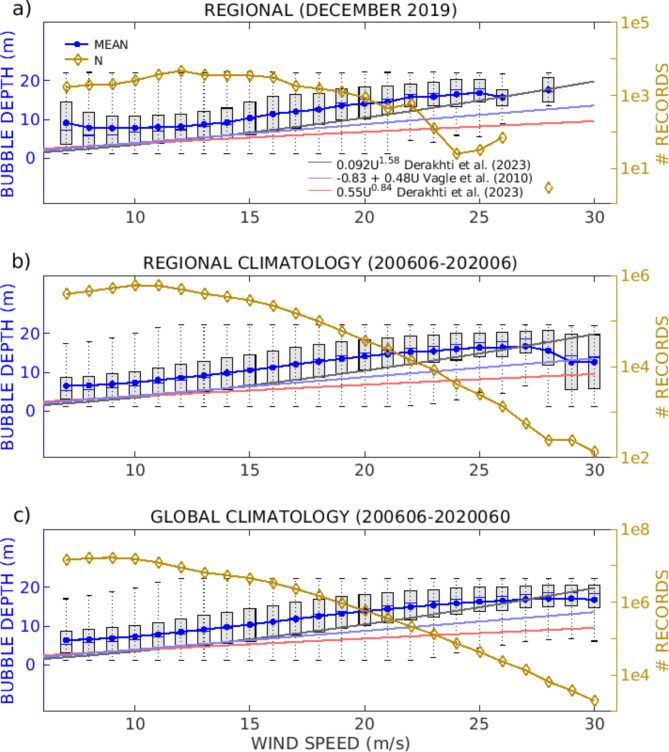
Bubble depth as a function of wind speed. (a) December 2019 in the Gulf of Alaska (blue); (b) 2006–2020 climatology in the Gulf of Alaska; (c) 2006–2020 climatology at global scale. The 3 curves are the three relationships discussed in^31^ Cifuentes-Lorenzen (2023). In all panels N is the number of data points (yellow curve with diamonds).

Figure 2a shows the CALIPSO bubble depth as a function of wind speed for all data collected in the Gulf of Alaska during December 2019. We extended the dataset temporal scale by using data from June 2006 to June 2020 (Fig. 2b). We then extended to the spatial scale by using data for the same time period but for the global oceans (Fig. 2c). The observed relationship between the bubble depth and wind speed in Fig. 2a seems to follow relatively well the various relationships presented in^31^. The best fit is with the mean plume depth of^13^, where there is a good agreement with the CALIPSO bubble depths above 25 m and a positive bias for the depths below 25 m. The variations in the bubble depth to wind speed relationships used in our comparison are expected and can be related to the ocean’s complexity. That being said, even if the agreement with^13^ is slightly better when we limit the data to the Gulf of Alaska and December 2019 (Fig. 2a), the trend for depths lower than 25 m does not change much when we extend the temporal (Fig. 2b) or the spatial scales (Fig. 2c); i.e., the bubble depth from CALIPSO stay biased high in all cases. If the relationship of^13^ is more representative of ocean physics than the other relationships, then the better agreement with the bubble depth observations with this relationship in December 2019 and at high wind speed would be consistent with the crosstalk issue in the space lidar. In 2019, the crosstalk contamination was lower than in the 2006–2020 dataset^32^. In addition, when the depolarization ratio is low, the crosstalk relative contribution is large. When the depolarization ratio is larger, the crosstalk can be neglected, which is the case for deep bubble clouds. Instrumental artifacts in the space lidar could explain why, when the depth is above 25 m, the CALIPSO bubble depth agrees much better with^13^.

Results from these satellite evaluations demonstrate the capability of CALIPSO to quantitatively derive the bubble depth values within the range of variability associated with echo sounders or shipboard lidar data. The data are similar to those of^31^, even if they are primarily biased high. Considering the novelty of the CALIPSO lidar bubble depth retrieval, it would make sense that the difference with the observations of^13^ comes from the inaccuracy of our first algorithm. However, the results are promising, and this justifies additional discussion of the CALIPSO bubble depth data at different scales.

### Comparison of CALIPSO bubble depth and AMSR2/WindSat whitecap fraction in the Gulf of Alaska

Breaking waves create bubbles, and this typically correlates with higher wind speed. CALIPSO depolarization increases as wind speed increases. Accordingly, the global open-ocean bubble depth structures should be qualitatively similar to the wind speed and whitecaps fraction measured from satellites.

**Fig. 3:**
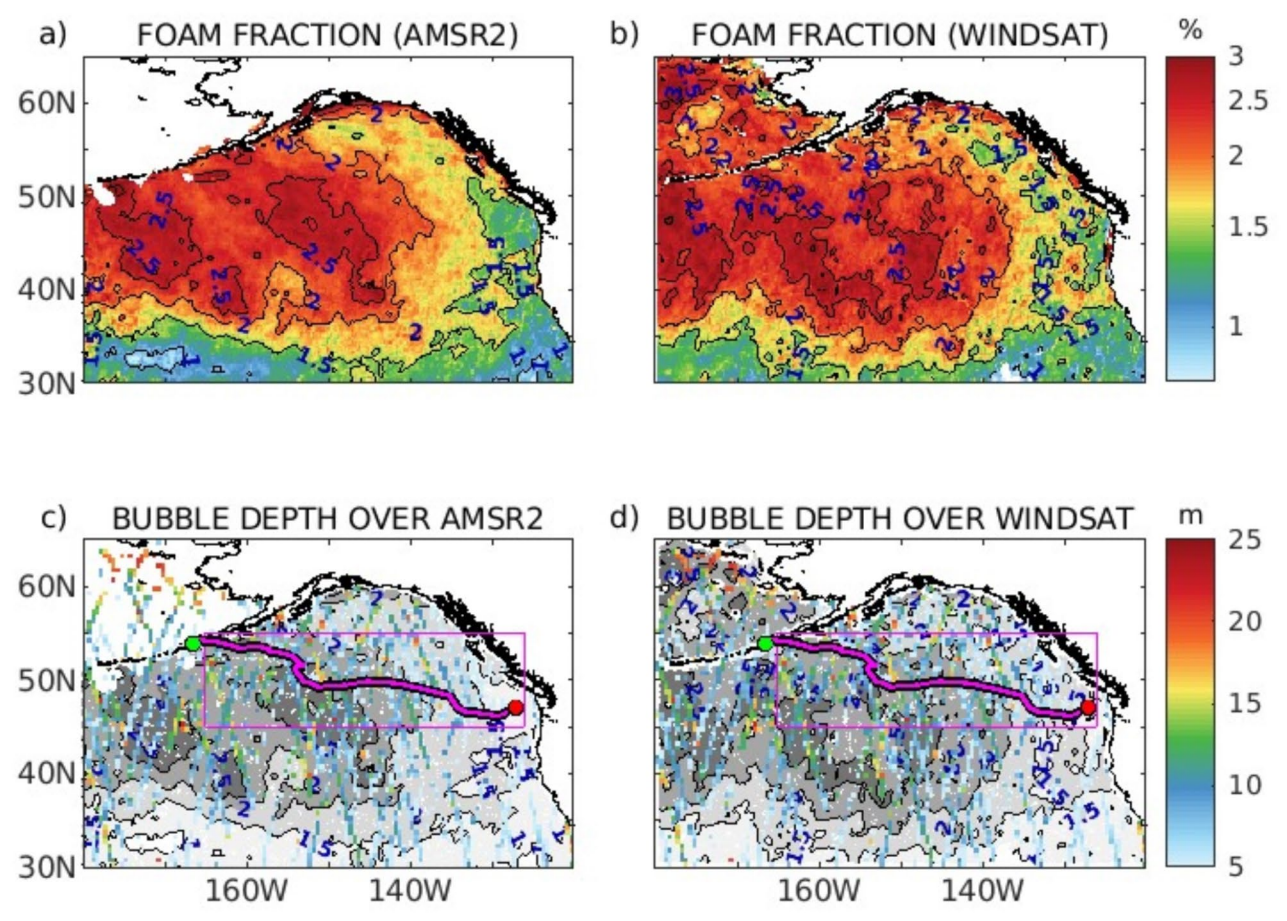
(a) Whitecap fraction from AMSR2 and (b) WindSat. (c) CALIPSO bubble depth with the countour of the whitecap fraction for AMSR2 and (d) WindSat. Only wind speed > 7 m/s are used in the analysis.

In Fig. 3, we show the similarities between the whitecap fraction from AMSR2 and WindSat in the Gulf of Alaska in December 2019 and the bubble depth of CALIPSO in the same area. As we can see, the contours of the higher whitecap fraction (Fig. 3c and d) show similarities with the highest values of the bubble depth.

Both the bubble depth and whitecap fraction are lower in the eastern and southern parts of the Gulf of Alaska. These variables increase in the northern and western parts of the Gulf. There are no data for AMSR2 for the northwestern part of this map, but the higher bubble depth in this area corresponds well to the WindSat higher whitecap fraction.

### Global CALIPSO bubble depth

At the global scale, we expect to observe strong seasonal cycles in bubble depth in the high latitudes (roaring sixties), where the higher wind speed significantly enhances breaking wave processes. This spatial and seasonal variability in wave breaking should be apparent in global patterns of bubble depth.

**Fig. 4:**
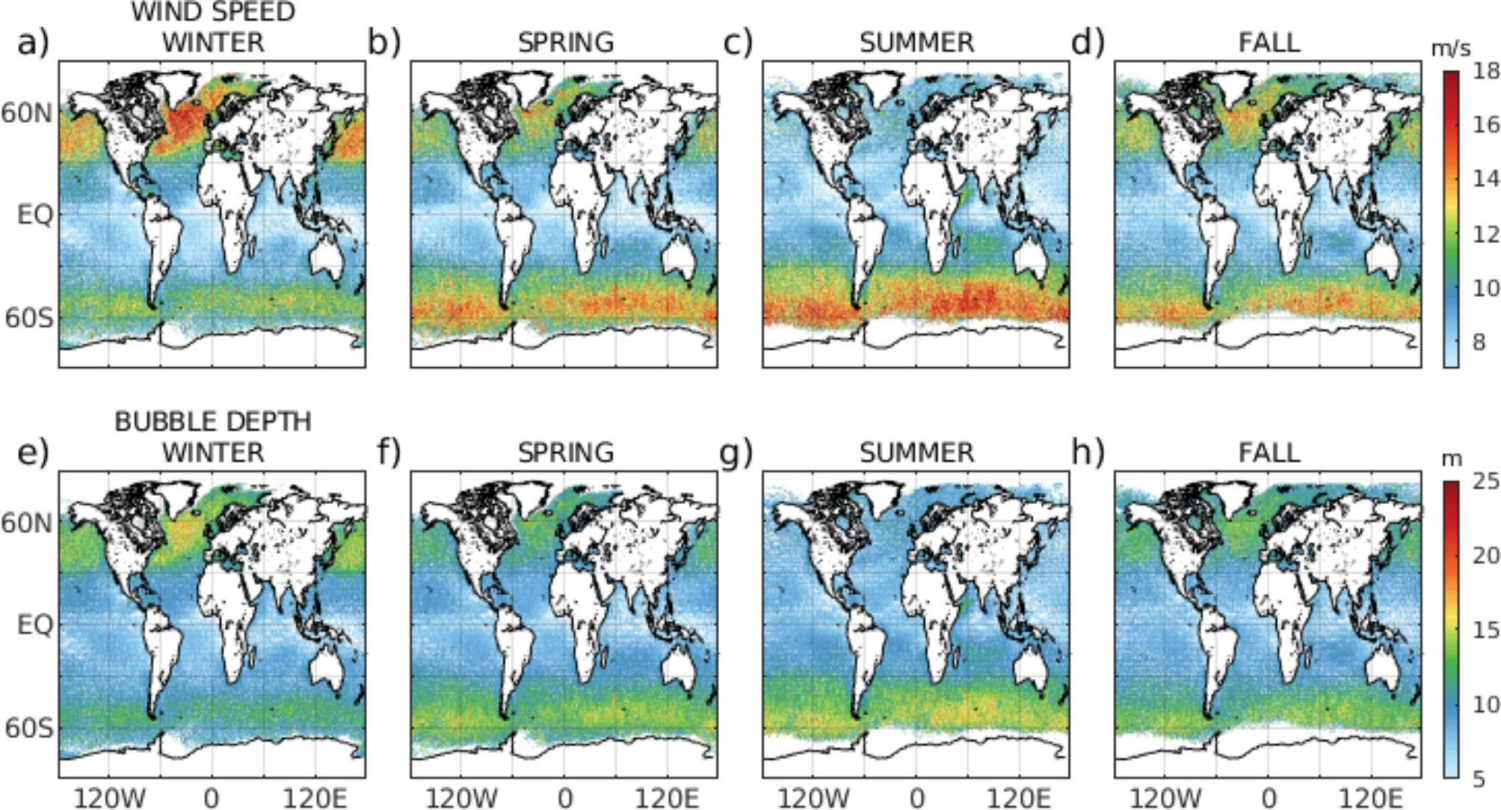
Seasonal maps of CALIPSO bubbles depths.

Combining all CALIPSO bubble depth data for June 2006–2020 analysis period yields a global climatology that exhibits all the anticipated major breaking wave features (Fig. 4). Elevated bubble depth values in the Southern Ocean reflect the region’s high wind speed while lower average values are found in the equator and tropics for all seasons.

Patchy seasonal variations in bubble depth in the Greenland, Iceland, and the United Kingdom gap are also reflected in the CALIPSO bubble depth data and correspond to varying sources of breaking waves.

Climatologies of bubble depth data for the Boreal summer (June–August) (Fig. 4c and g) and Boreal winter (December–February) (Fig. 4a and e) further illustrate the strong seasonality of high-latitude bubble depth and, again, demonstrate the feasibility of characterizing underwater bubble depth and their variability with a space-based lidar.

## Conclusions

The results presented here demonstrate the quantitative measurement of bubble depth with a space-based lidar. CALIPSO bubble depth retrievals allow independent assessments of missing energy and provide a globally comprehensive data set for algorithm development of bubble depth to whitecap fraction algorithm, thus addressing a key knowledge gap in ocean physics. The next step is to combine the space lidar bubble depth with AMSR2 whitecap fraction to directly measure the three-dimensional bubble properties globally.

## Supplementary Information

Supplementary Information.

## Data Availability

The SODA, AMSR-E/AMSR2 and CALIPSO data used in this research are publicly available (after registration to https://www.icare.univ-lille.fr/asd-content/register, go to https://www.icare.univ-lille.fr/asd-content/archive/?dir=CALIOP/SODA-333m.v2.30/ and https://www.icare.univ-lille.fr/asd-content/archive/?dir=CALIOP/SODA-333m.v2.31/) AMSR2 brightness temperature data used for whitecap fraction retrievals were downloaded from JAXA (https://www.eorc.jaxa.jp/AMSR/datacatalog/tb/). All the other data used in this study are archived at (https://data.mendeley.com/datasets/scjcjyvjhn/1) with (DOI: 10.17632/scjcjyvjhn.1).
