# Supplementary material for: Using space lidar to infer bubble cloud depth on a global scale
Source: Sci Rep. 2024 Oct 18;14:24525. doi: 10.1038/s41598-024-75551-6 (PMC11489409; doi:10.1038/s41598-024-75551-6)
Supplement: Supplementary file 1 — Supplementary Information. [file 41598_2024_75551_MOESM1_ESM.docx]

**Addendum for Scientific Reports**

Global scale measurement of Ocean Bubble Depth with Space Lidar

Damien Josset^1^*, Stephanie Cayula^1^, Magdalena Anguelova^2^, Erick Rogers^1^, David Wang^1^

^1^U.S. Naval Research Laboratory, NASA Stennis Space Center, MS 39529, USA

*(email) damien.b.josset.civ@us.navy.mil (phone: 228-688-4408)

^2^U.S. Naval Research Laboratory, Washington, DC 20375, USA

**Method M1.** Detailed description of CALIPSO data analysis and product uncertainties.

**Figure M.1.** Bubble depth to depolarization relationship.

**Figure M.2.** Histogram of the occurrences of depolarization from CALIPSO and the NRL shipboard lidar.

**Figure M.3.** Histogram of the occurrences of CALIPSO bubble depth observations in the Gulf of Alaska for one Month (December 2019).

**Figure M.4**. Measurement geometry of the space lidar.

**Figure M.5.** Ratio of the apparent bubble depth δ_B_ measured by CALIPSO for a specific bubble cloud to the maximum depolarization at the maximum depth.

**Figure M.6.** Bias between our data and the relationship of (Vagle et al. 2010).

**Method M1. Detailed description of CALIPSO data analysis and product uncertainties.**

1. **Analysis of CALIPSO data**

In theory, the bubble clouds have a signature in the co-polarization lidar channel (Churnside 2010). This is observed in very clean ocean water in the laboratory (Wang et al. 2022) and as a statistical monthly average (Josset et al. 2010). However, in practice, the bubble signature is too small in the co-polarization channel, and it is only feasible to discriminate the individual bubble clouds from the ocean background in natural conditions with another set of information like the depolarization (Josset et al. 2024).

The signature of the ocean bubbles in the depolarization channel is strong and unambiguous (Hu et al. 2008, Churnside 2014, Wang et al. 2022, Josset et al. 2024). This change of depolarization is much higher than for most features typically observed by an ocean lidar (water molecules, phytoplankton). It has been demonstrated that phytoplankton can be detected by a space lidar (Behrenfeld et al., 2013), so there is no conceptual difficulty in detecting underwater bubbles from space as well.

However, the CALIPSO lidar vertical sampling resolution is relatively coarse. At the top of the ocean, the resolution is related to the 10 MHz digitizer rate (Hunt et al. 2009) and onboard averaging of two vertical bins (which leads to a resolution of 29.98 m in air). The ocean water refractive index is around 1.338 at 532 nm (Quan and Fry, 1995), so the speed of light in this medium is reduced accordingly, and the vertical sampling of the ocean's upper column is 22.4 m. Although the deepest bubble clouds reach beyond this value (Strand et al. 2020, Derakhti et al. 2023, Josset et al. 2024), the number of occurrences of bubble clouds decreases non-linearly as a function of their depth. Most observations are in depth ranging from 0 to 15 m (Fig. M.1). Therefore, for most bubble clouds, the space lidar does not have a vertical resolution sufficient to derive directly the bubble depth from the profile information.

Our estimates of bubble depth are based on our shipboard lidar field observations (Josset et al. 2024). The lidar was on the bow of the R/V Sikuliaq from 4^th^ December 2019 to 23^rd^ December 2019. The average wind speed value was 9.7 m/s (± 4.8 m/s), with a minimum of 0.05 and a maximum of 26.3 m/s (with wind gusts up to 32.9 m/s). Wave heights ranged from 3 to 10 m, with extreme wave events in the area as recorded by the surface wave instrument floats with tracking (SWIFT) buoys (Thomson et al. 2012, 2019) up to 17 m. We collected more than 113 hours of data at 50 Hz (around 20×10^6^ ocean profiles) that span different wind and wave conditions. This lidar dataset is presented in more detail in Josset et al. (2024)

The shipboard lidar can derive the bubble cloud with a high vertical resolution (14 cm), which provides guidance for understanding coarser data. Using all the data from this unique field experiment, we found a clear relationship between the column-integrated backscatter depolarization from the ocean and the maximum bubble depth detected in the lidar profiles (Fig. M.1). The vertical range corresponding to the shipboard lidar “column-integrated” depolarization ratio goes from 0.05 m above to 32.8 m below the detected surface.

The advantage of using the integrated depolarization ratio is that we do not use the vertical information, and the column-integrated ratio of the cross-polarized to co-polarized channel is the most accurate measurement of the CALIPSO lidar in terms of calibration. The Vertical Feature Mask (VFM) was used to filter out scenes with liquid water clouds, ice clouds, and stratospheric features. The VFM filtering method removes data with a low signal-to-noise ratio, even if the calibration accuracy should be insensitive to the feature attenuation. The depolarization ratio $\delta_{R}$ is the ratio of the perpendicular channel $C_{\perp}$ (cross-polarization) to the parallel channel (co-polarization) $C_{\parallel}$:

$\delta_{R}=\frac{C_{\perp}}{C_{\parallel}}$ (1)

This quantity exists for both the shipboard lidar and the space lidar. For this first algorithm, we expressed the bubble depth $D_{B}$as a function of $\delta_{R}$ through the following relationship:

$D_{B}=-20.18\left( \delta_{R}^{-0.255} \right)+58.47$ (2)

The challenge is to estimate the accuracy of the relationship between the bubble depth and the depolarization ratio for a different instrument. We anticipate the uncertainty to decrease with future versions of the algorithm as we learn more about this new retrieval methodology and obtain more data for validation. Three sources of uncertainty will modify the relationship between the shipboard and space lidar. First, the laser footprint is around 80 m (Hostetler et al., 2006). Even when a bubble cloud is present, the signal will likely include a decent amount of return from water molecules, and the depolarization will be smaller than if only bubbles were present. Second, the amount of multiple scattering increases the light depolarization (Xu and Alfano, 2005). Third, the crosstalk in the space lidar data is between 0.3% and 0.7% between 2006 and 2020 (Lu et al. 2021). We expect the crosstalk to be around 0.2-0.3% in 2019 in the Gulf of Alaska, and around 0.4% when we discuss the 2006-2020 dataset.

CALIPSO keeps a higher amount of multiple scattered light in its receiver field of view than the shipboard lidar and the distribution of depolarization is shifted towards higher values (Fig. M.2). For the data in the Gulf of Alaska, the median depolarization of the shipboard observations is 0.0196 while it is 0.0329 for CALIPSO. Fig. M.2 shows the medians and the quartiles. As we can see, 75% of the depolarization observations for the shipboard lidar are below 0.025, and for CALIPSO, 75% are below 0.0534.

**(b) Uncertainties in Satellite Retrieved Products**

The sources of uncertainty for the CALIPSO bubble depth estimates include errors related to the instrument calibration. It also includes errors associated with assumptions regarding the similarities of measurement geometry and scattering properties between the shipboard lidar and the space lidar.

In CALIPSO, the ratio between the parallel and perpendicular detector is internally calibrated with a pseudo-depolarizer using a specific maneuver. Consequently, the random error in the depolarization ratio measurements has remained at ∼1% or less (Powell et al. 2009; Behrenfeld et al. 2013). The final random noise uncertainty depends on the spatial averaging of the CALIPSO depolarization ratio measurements. The CALIOP layer-integrated backscatter data reported in the current study are monthly and seasonal average values within 0.5° X 0.5° pixels. For the monthly average in the Gulf of Alaska, we represent the statistic of observations in Fig. M.3. The grid is 71x122 pixels (8591 pixels). For the 1764 pixels that contain data, the median of the counts per pixel is 12 (minimum of 1, maximum of 168). The mapping process typically reduces the random noise to less than 0.3%.

The bias (systematic errors) in CALIPSO depolarization ratio measurements of the bubbles is in the order of 4 to 6% during nighttime in the version of the level 1 data prior to V4.51 (Vaughan et al., 2023). Neglecting crosstalk contribution, we expect this bias to be less than 1.5% (Powell et al. 2009, Behrenfeld et al. 2013) for analysis based on V4.51 and subsequent versions.

The version of the SODA used for this paper (V2-30 and V2-31) is based on CALIPSO level 1, V4.10, and level 2, V4.20. The multi-instrumental nature of SODA is not conducive to frequent reprocessing. However, a final reprocessing of SODA is scheduled with CALIPSO V5. Overall, the error related to the instrument calibration based on both nighttime and daytime data should be less than 5%, and less than 2% for the next reprocessing (with SODA V3 based on CALIPSO V5).

The viewing geometry is illustrated in Fig. M.4. The shipboard lidar provides high-resolution observations that can resolve the shape of the bubble cloud. The shape can be approximated as a cone (Novarini et al. 1998, Josset et al. 2024). The space lidar, however, measures a cylinder of water, and a fraction of this cylinder will contain bubbles.

What is important to define in this context is the depolarization in a bubble cloud $\delta_{B}$, weighted by the volume of bubbles $V_{B}$

$\delta_{B}=\frac{\int_{0}^{R} dV_{B}(r)\delta_{R}(r)dr}{V_{B}(R)}$ (3)

The volume of the bubble cloud $V_{B}$ is assumed to be a cone with a maximum depth related to the maximum depolarization and is given by (2). The radius of this bubble cone is estimated based on the relationship of Derakhti et al. (2023) that relates the whitecaps fraction to the bubble penetration depth. We convert this relationship to a bubble radius $R_{B}$, as the radius of the CALIPSO laser footprint is fixed and determines the observation geometry. In this equation, it is assumed that the bubble cloud structure is one circle at the ocean surface.

$R_{B}=\left[ 40\sqrt{\frac{D_{B}}{29.5}} \right]^{3.0303}$ (4)

Through (4), we can find the apparent depolarization measured by CALIPSO for a bubble cloud of this geometry. This is shown in Fig. M.5 (Left). Figure M.5 (Right) also illustrates the relative value of the bubble depth if the space lidar measured this depolarization instead of the maximum depolarization. The error is a low bias of around 20%.

However, this determination is inconsistent with the increase of depolarization apparent in CALIPSO observations (Fig. M.2). So far, we neglected the presence of non-bubbly water in the laser footprint and the presence of multiple scattering. We provide partial answers on quantifying the presence of multiple scattering in the following text, but we would need more information to determine the theoretical expectation. However, we can determine the total bias from the measurements themselves and quantify the impact of multiple scattering on the space lidar observations. This discussion and the bias derivation will hopefully guide future work to better determine the space lidar uncertainty as well as optimize future ocean lidar designs.

The apparent depolarization $\delta_{A}$ within the CALIPSO footprint is the weighted average between the depolarization of the bubble cloud and the depolarization of the water without bubbles ($\delta_{W}$) for a given volume of water $V_{W}$; it can be written as:

$\delta_{A}=\frac{V_{B}\delta_{B}+V_{W}\delta_{W}}{V_{T}}$ (5)

Equation (5) assumes that the co-polarized signal does not change in presence of bubbles. It is not accurate (Hu et al. 2008, Josset et al. 2010), but it should be a valid approximation when the amount of bubble is low. When there are a lot of bubbles, then $\delta_{A}$ becomes closer to $\delta_{B}$.

Depending on the exact penetration depth of the space lidar, the theoretical uncertainty determination will vary because the ratio of water with or without bubbles changes.

Based on measurements on the R/V Sikuliaq, when the water is clear, the NRL shipboard lidar seems to have a penetration depth limited to around 12 m. Note that the penetration depth may increase when features are present in the water column (bubbles, phytoplankton layers, etc.), and the maximum bubble depth measured was 30.93 m. The signal of bubbles detected by the NRL shipboard lidar falls under the small-angle multiple scattering regime (Josset et al. 2024), and based on the dataset we gathered in the Gulf of Alaska, bubbles are an ideal target for an ocean lidar (large scattering signal, low absorption). A thick layer of phytoplankton and sediments typically reduces the penetration depth. However, a well-designed ocean lidar can still detect the presence of an isolated layer at a larger depth than expected when only water molecules are above it.

It is difficult to estimate how deep a space lidar like CALIPSO would penetrate in similar circumstances because the details of the hardware matter considerably. For example, the signal output would be identical for the shipboard lidar at 12 m and CALIPSO at 3 m when we make the following assumptions: (i) We can neglect multiple scattering; (ii) The hardware of the shipboard and space lidars have similar efficiency; (iii) The only differences are the signal strength (the ship board lidar has a higher signal by around 7 orders of magnitude) and the dynamic range (CALIPSO has a higher dynamic range by around 3 orders of magnitude); and (iv) CALIPSO noise level is twice the electronic noise of the shipboard lidar (when neglecting the sun noise). This determination is approximate, and the CALIPSO penetration could be much deeper in all cases because of multiple scattering, especially when a phytoplankton layer is present (Churnside et al., 2013).

Theoretically, the exact amount of multiple scattering in the CALIPSO lidar underwater measurements is challenging to evaluate. Such calculations are typically approximate, and, for a homogeneous medium, it requires a complete knowledge of the scattering properties. The presence of two media (water and bubbles) further complicates this determination. However, based on the statistics of the data shown in Fig. M.2 and the difference between the CALIPSO bubble depth and the relationship between bubble depth and wind speed derived by Vagle et al. (2010), the bubble depth is biased by around 4.5 m. Fig. M.6 (Left) shows the estimated bias in the Gulf of Alaska for December 2019. This bias allows us to estimate the increase in depolarization associated with it (shown in Fig M.6 Right). This estimated increase of the depolarization due to wide-angle multiple scattering goes from around 1.2 for the smallest bubble clouds around wind speed of 10 m/s to around 1.9 for the largest bubble clouds. Although it is difficult to estimate what the multiple scattering should be theoretically, these numbers are consistent with a relatively low amount of multiple scattering when there are not many bubbles in the water and a higher increase when there are more bubbles in the water. A doubling of the depolarization at high wind speed seems consistent with lidar multiple scattering in dense medium (Hu et al. 2006, Hogan et al. 2008).

In terms of random error, all the instrumental errors we discussed can be neglected. However, the dispersion of the depolarization to depth relationship is an important source of uncertainty, probably due to the geophysical variability of the bubble clouds. The standard deviation of the data in Fig. M.1 has a median of 2.5 m between 0.02 and 0.038, where there is enough data to calculate the statistic.

Overall, the error of our bubble depth estimate is 4.5 m ± 2.5 m with this initial version of the bubble depth algorithm. This is an important information, and we anticipate to be able to reduce this error in the future.

Future improvements of the bubble depth algorithm related to the crosstalk and Polarization Gain Ration corrections will change the final bias. Assuming a crosstalk of around 0.4% applies to this analysis, a bubble depth retrieval of 10.27 m corresponding to a depolarization ratio of 0.0329, will after crosstalk correction become a bubble depth of 8.59 m with a depolarization ratio of 0.0288. The bubble depth difference due to the crosstalk here is 1.68 m. This is a considerable reduction of the bias that we’ll need to consider in the next version of the algorithm.

Note that other particles in the ocean, such as phytoplankton, also depolarize the laser light. In this study, we only took this contribution into account statistically. The 2006-2020 average depolarization of phytoplankton at global scale and for low wind speed is (around 0.007 and) lower than the lowest depolarization we consider valid for bubbles (0.015). Although we would expect the uncertainty estimate to be higher when the wind speed is low, we do not notice an increase of the bias in Fig. 2 at global scale or regionally for an extended dataset (b and c). However, there is a notable increase in the bias at low wind speed in the Gulf of Alaska in December 2019 (Fig. 2a). Fig. 2 would seem to confirm that our methodology works in the statistical sense (as it should by design), but that the bias can be expected to increase in specific areas even if they are moderately productive. Based on in-situ measurements during our field mission in 2019, the chlorophyll-a content was relatively stable at 1.94 ± 0.32 mg.m^−3^. A possible way to correct this error source is to determine the depolarization at low wind speed first, then correct this baseline before we use the bubble depth retrieval algorithm. We will explore this possibility in the next version of the algorithm.

**References:**

Behrenfeld, M. J., Y. Hu, C.A. Hostetler, G. Dall'Olmo, S.D. Rodier, J.W. Hair, C.R. Trepte (2013). Space-based lidar measurements of global ocean carbon stocks, Geophys. Res. Lett., 40 , pp. 4355-4360, [10.1002/grl.50816](https://doi.org/10.1002/grl.50816)

Churnside, J. H. (2010). Lidar signature from bubbles in the sea, Vol. 18, No. 8/OPTICS EXPRESS

Churnside, J. H.; McCarty, B.J.; Lu, X. (2013). Subsurface Ocean Signals from an Orbiting Polarization Lidar. Remote Sens., 5, 3457-3475. https://doi.org/10.3390/rs5073457.

Churnside, J. H. (2014). Review of profiling oceanographic lidar, Optical Engineering, 56(7), 1, DOI : <https://doi.org/10.1117/1.oe.56.7.079802>.

Derakhti, Morteza, Jim Thomson, Christopher S Bassett, et al. (2023). Statistics of bubble plumes generated by breaking surface waves. *ESS Open Archive.* February 27, DOI: [10.22541/essoar.167751591.11265648/v1](https://doi.org/10.22541/essoar.167751591.11265648/v1)

Hogan, R. J., and Battaglia, A. (2008). Fast Lidar and Radar Multiple-Scattering Models. Part II: Wide-Angle Scattering Using the Time-Dependent Two-Stream Approximation. *J. Atmos. Sci.*, **65**, 3636–3651, <https://doi.org/10.1175/2008JAS2643.1>.

C. A. Hostetler, Z. Liu, J. Reagan, M. Vaughan, D. Winker, M. Osborn, W. H. Hunt, K. A. Powell, and C. Trepte. CALIOP Algorithm Theoretical Basis Document : Calibration and level 1 data products, 2006.

Yongxiang, Hu, Zhaoyan Liu, David Winker, Mark Vaughan, Vincent Noel, Luc Bissonnette, Gilles Roy, and Matthew McGill, (2006). Simple relation between lidar multiple scattering and depolarization for water clouds, Opt. Lett. **31**, 1809-1811.

Hu, Y., Stamnes, K., Vaughan, M., Pelon, J., Weimer, C., Wu, D., Cisewski, M., Sun, W., Yang, P., Lin, B., Omar, A., Flittner, D., Hostetler, C., Trepte, C., Winker, D., Gibson, G., and Santa-Maria, M. (2008). Sea surface wind speed estimation from space-based lidar measurements, Atmos. Chem. Phys., 8, 3593–3601, https://doi.org/10.5194/acp-8-3593-2008.

Hunt, W. H., D. M. Winker, M. A. Vaughan, K. A. Powell, P. L. Lucker, and C. Weimer, (2009). CALIPSO Lidar Description and Performance Assessment. *J. Atmos. Oceanic Technol.*, **26**, 1214–1228, <https://doi.org/10.1175/2009JTECHA1223.1>.

Josset, D., J. Pelon and Hu, Y. (2010). Multi-Instrument Calibration Method Based on a Multiwavelength Ocean Surface Model, in IEEE Geoscience and Remote Sensing Letters, vol. 7, no. 1, pp. 195-199, doi: 10.1109/LGRS.2009.2030906.

D. Josset, S. Cayula, B. Concannon, S. Sova, and A. Weidemann, "On the bubble-bubbleless ocean continuum and its meaning for the LiDAR equation: LiDAR measurement of underwater bubble properties during storm conditions," Opt. Express **32**, 20881-20903 (2024).

Lu, X.; Hu, Y.; Omar, A.; Baize, R.; Vaughan, M.; Rodier, S.; Kar, J.; Getzewich, B.; Lucker, P.; Trepte, C.; et al. Global Ocean Studies from CALIOP/CALIPSO by Removing Polarization Crosstalk Effects. Remote Sens. **2021**, 13, 2769. <https://doi.org/> 10.3390/rs13142769.

Novarini, J. C. , R. S. Keiffer and G. V. Norton, (1998). A model for variations in the range and depth dependence of the sound speed and attenuation induced by bubble clouds under wind-driven sea surfaces, in IEEE Journal of Oceanic Engineering, vol. 23, no. 4, pp. 423-438, doi: 10.1109/48.725236.

Powell, K. A., and Coauthors, (2009). CALIPSO Lidar Calibration Algorithms. Part I: Nighttime 532-nm Parallel Channel and 532-nm Perpendicular Channel. *J. Atmos. Oceanic Technol.*, **26**, 2015–2033, <https://doi.org/10.1175/2009JTECHA1242.1>.

Quan, Xiaohong and Edward S. Fry, (1995). Empirical equation for the index of refraction of seawater, Appl. Opt. **34**, 3477-3480. doi: [10.1364/AO.34.003477](https://doi.org/10.1364/AO.34.003477)

Strand, K. O., O. Breivik, G. Pedersen, F. B. Vikebo, S. Sundby, and K. H. Christensen (2020). Long-term statistics of observed bubble depth versus modeled wave dissipation, J. Geophys. Res.: Oceans, 125, e2019JC015,906, doi:10.1029/2019JC015906.

Thomson, J., (2012). Wave Breaking Dissipation Observed with “SWIFT” Drifters, Journal of Atmospheric and Oceanic Technology, 29(12), 1866-1882.

Thomson, J., et al. (2019). A new version of the SWIFT platform for waves, currents, and turbulence in the ocean surface layer, 2019 IEEE/OES Twelfth Current, Waves and Turbulence Measurement (CWTM), pp. 1-7, doi: 10.1109/CWTM43797.2019.8955299.

Vagle, S., McNeil, C., & Steiner, N. (2010). Upper ocean bubble measurements from the NE Pacific and estimates of their role in air-sea gas transfer of the weakly soluble gases nitrogen and oxygen. *Journal of Geophysical Research*, *115*(C12), C12054., <https://doi.org/10.1029/2009JC005990>

Vaughan, M., S. D. Rodier, Z. Liu, A. Garnier, K.-P. Lee, B. Getzewich and S. Zeng, 2023: “Correcting CALIOP Polarization Gain Ratios for Diurnal Variations”, in Proceedings of the 30th International Laser Radar Conference, Sullivan, J. T., T. Leblanc, S. Tucker, B. Demoz, E. Eloranta, C. Hostetler, S. Ishii, L. Mona, F. Moshary, A. Papayannis and K. Rupavatharam, Eds., pp. 691–697, Springer Atmospheric Sciences, Springer.

Wang, D.; Josset, D.; Savelyev, I.; Anguelova, M.; Cayula, S.; Abelev, A. An Experimental Study on Measuring Breaking-Wave Bubbles with LiDAR Remote Sensing. Remote Sens. 2022, 14, 1680. https://doi.org/10.3390/rs14071680.

M. Xu and R. R. Alfano, “Random walk of polarized light in turbid media,” Phys. Rev. Lett. 95(21), 213901 (2005).


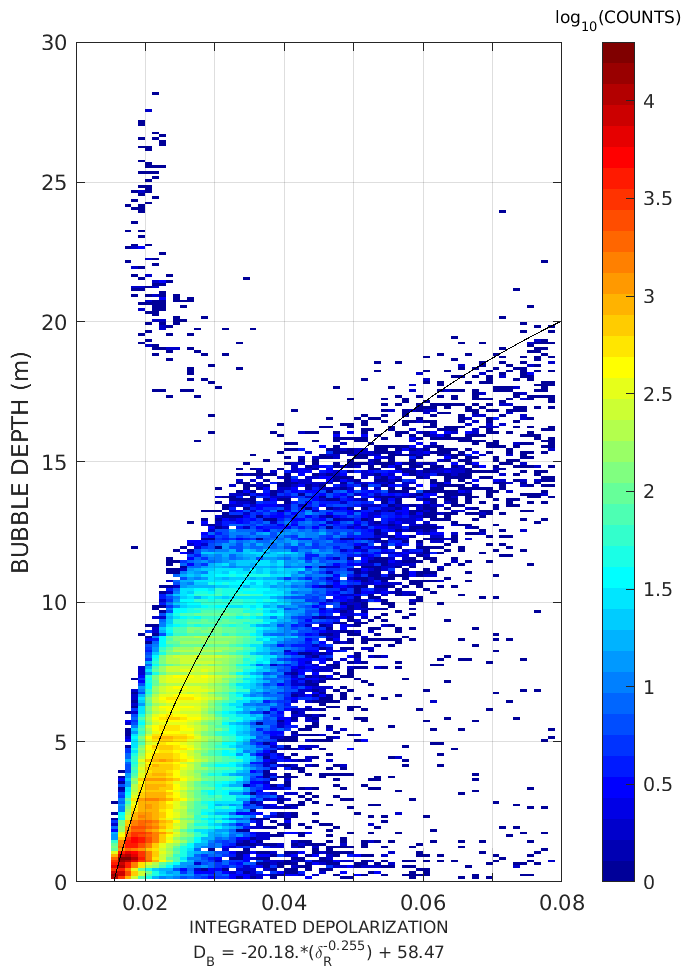


Figure M.1. Illustration of the relationship between integrated depolarization and the bubble depth for wind speed higher than 7 m/s. The color bar shows the decimal logarithm of the number of observations.


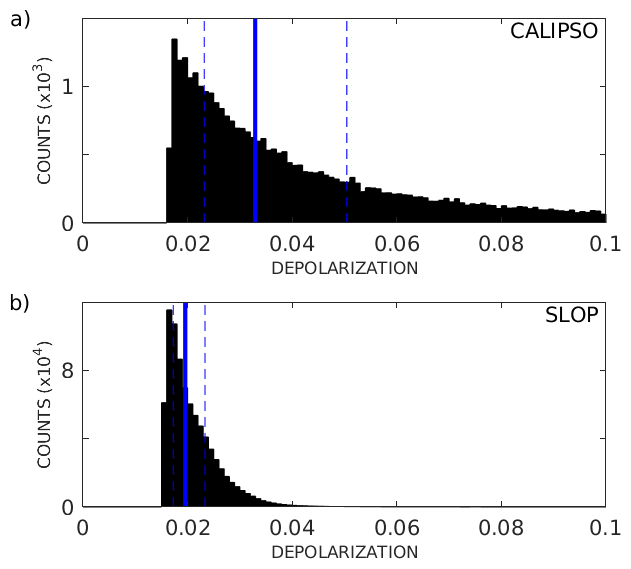


Figure M.2. Histogram of the occurrences of depolarization from CALIPSO (Top) and the NRL shipboard lidar (Bottom). The blue vertical solid line is the median. The two light blue vertical dotted lines define one standard deviation around the median.


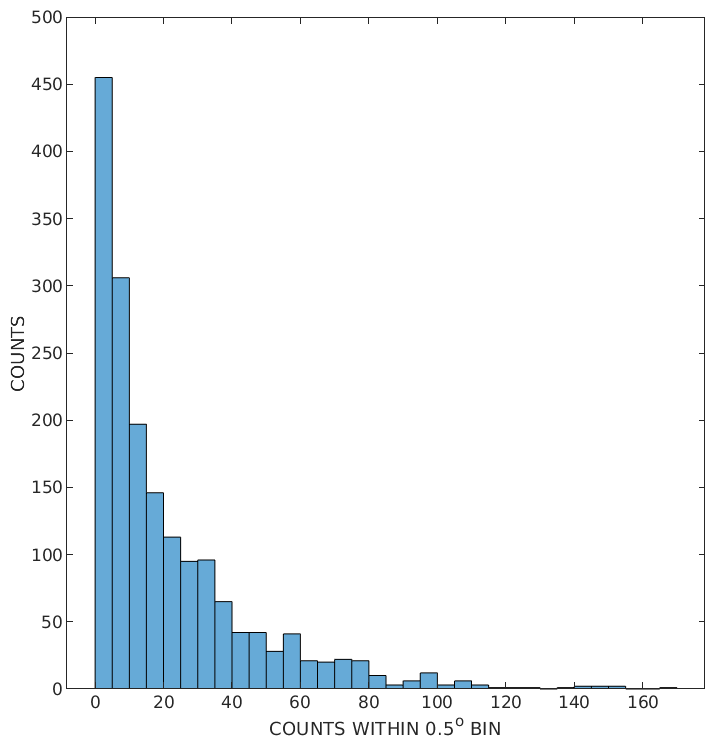


Figure M.3. Histogram of the occurrences of CALIPSO bubble depth observations in the Gulf of Alaska for one Month (December 2019).


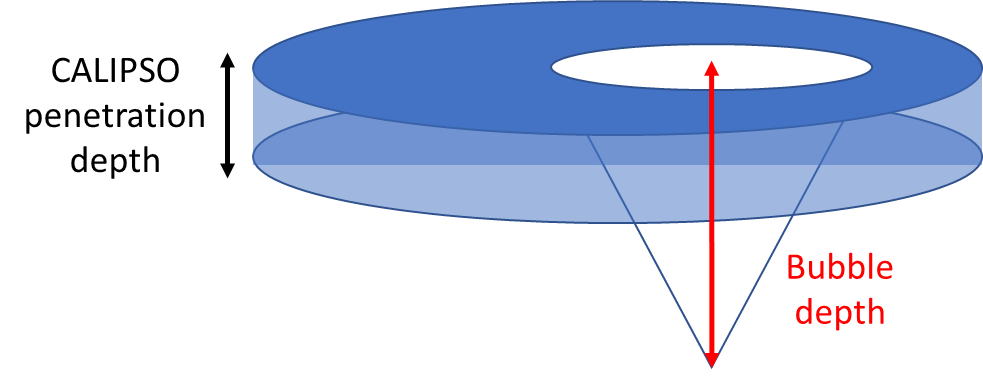


Figure M.4. Measurement geometry of the space lidar.


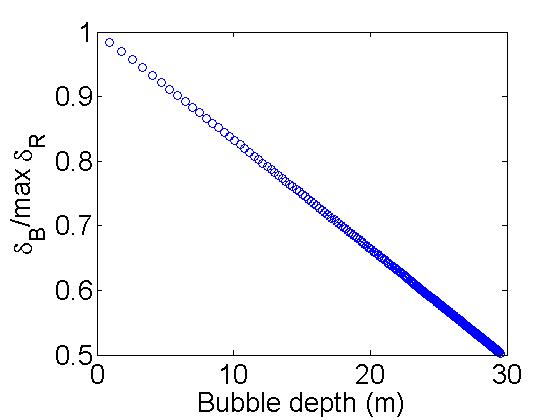

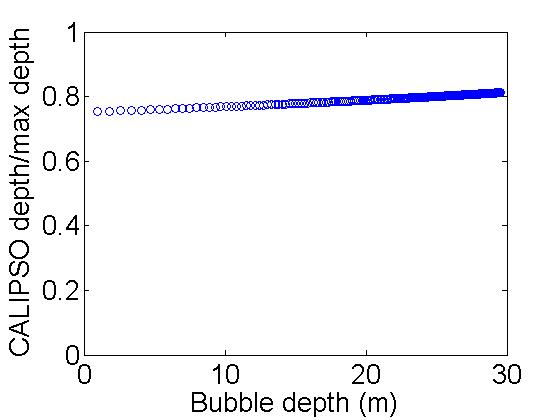


Figure M.5. Left: Neglecting multiple scattering, theoretical ratio of the bubble depolarization $\delta_{B}$ measured by CALIPSO for a specific bubble cloud to the maximum depolarization at the maximum depth, as a function of this maximum depth. Right: Same picture for the measured depth as a function of the maximum depth.


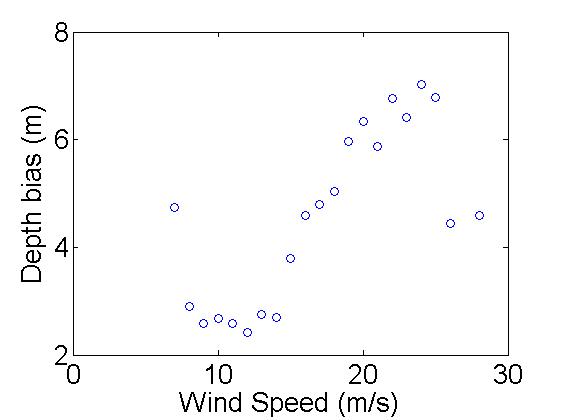

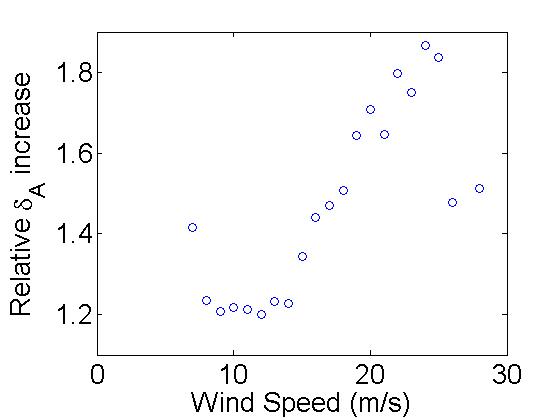


Figure M.6. Left: Bias between our data and the relationship of Vagle et al. (2010). Right: Relative increase of apparent depolarization $\delta_{A}$ due to the multiple scattering and other error sources based on the bias shown on the left.
